# Antimalarial Activity of Aqueous and 80% Methanol Crude Seed Extracts and Solvent Fractions of *Schinus molle* Linnaeus (*Anacardiaceae*) in *Plasmodium berghei*-Infected Mice

**DOI:** 10.1155/2020/9473250

**Published:** 2020-02-18

**Authors:** Getu Habte, Teshome Nedi, Solomon Assefa

**Affiliations:** ^1^Department of Pharmacology and Clinical Pharmacy, School of Pharmacy, College of Health Sciences, Addis Ababa University, P.O. Box 1176, Addis Ababa, Ethiopia; ^2^Department of Pharmacy, College of Health Sciences, Mettu University, P.O. Box 318, Addis Ababa, Ethiopia

## Abstract

**Background:**

Malaria is among the leading causes of mortality and morbidity. Moreover, the emergence of resistance to antimalarial drugs is a major problem in controlling the disease. This makes the development of novel antimalarial drugs a necessity. Medicinal plants are important sources in discovering antimalarial drugs. *Schinus molle* is claimed for its antimalarial effect in Ethiopian folkloric medicine and endowed with *in vitro* antiplasmodial activity. In the present study, the *in vivo* antimalarial activity of the plant was investigated.

**Methods:**

Acute toxicity was carried out using a standard procedure. To screen the *in vivo* antimalarial activity of the plant was investigated. *S. molle* against *Plasmodium berghei* (ANKA), a 4-day suppressive test was employed. The extracts and fractions were given to infected mice by oral gavage at 100, 200, and 400 mg/kg/day for four consecutive days. Parameters such as parasitemia were then evaluated.

**Results:**

Any sign of toxicity was not observed in the oral acute toxicity test. The crude extracts and solvent fractions exerted a significant (*p* < 0.05) inhibition of parasite load compared to the negative control. The highest inhibition (66.91%) was exhibited by the 400 mg/kg/day dose of 80% methanolic crude extract. Among the fractions, chloroform fraction demonstrated maximal chemosuppressive effect (55.60%). Moreover, crude extracts and solvent fractions prevented body weight loss, reduction in temperature, and anemia compared to the negative control. Except the aqueous fraction, the tested plant extracts were able to significantly prolong the survival time of infected mice.

**Conclusion:**

The findings of the present study confirmed the safety and a promising *in vivo* antimalarial activity of *S. molle*, thus supporting the traditional claim and *in vitro* efficacy. In-depth investigations on the plant, however, are highly recommended.*in vivo* antimalarial activity of the plant was investigated. *S. molle* against *in vitro* antiplasmodial activity. In the present study, the

## 1. Background

Malaria is a protozoal disease caused by parasites of the genus *Plasmodium* and primarily transmitted to human and other animals by a female anopheline mosquito [1, 2]. Besides, there are uncommon modes of malaria transmission such as congenital, blood transfusions, organ transplantation, and contaminated needles [3]. Five of the *Plasmodium* species, *P. falciparum*, *P. vivax*, *P. ovale*, *P. malariae*, and *P. knowlesi*, have been well known to cause human malaria [4, 5].

Globally, an estimated 3.2 billion people are at risk of being infected with malaria [6]. The burden is particularly the heaviest in the African region, where an estimated 90% of all malaria deaths occur [7]. Children aged less than 5 years account for 78% of all deaths [8]. Furthermore, pregnant women are highly vulnerable to malaria [6, 9]. In many parts of sub-Saharan Africa, the malaria eradication program, which was launched in the 1950s and 1960s, had little success [10]. Ethiopia, like other sub-Saharan African countries, shares the intolerable burden of malaria, which has become a leading public health problem in the country [3].

In developing countries, malaria is still the leading cause of outpatient visits and hospital admission. Development and spread of antimalarial drug resistance has been the major challenge towards the control of the disease [11, 12]. More importantly, there are alarming reports on parasite resistance to currently existing first-line drug regimen, ACTs [13]. This problem urges the search for new and improved antimalarial drugs [12, 14].

Medicinal plants played a crucial role as a source for antimalarial drugs including artemisinin and quinine [15, 16]. The seed of the experimental plant, *Schinus molle*, is used as folk medicine for treatment of malaria in different parts of Ethiopia [17–19]. The plant, moreover, possesses an *in vitro* antimalarial activity [20]. Consequently, based on the traditional claim and reported *in vitro* antimalarial activity, the present study evaluated the *in vivo* antimalarial activity of crude seed extracts and solvent fractions of *S. molle*.

## 2. Materials and Methods

### 2.1. Experimental Animals

In this study, healthy Swiss albino mice (weighing 25–31 g and 6–8 weeks of age), bred and maintained in the animal house of School of Pharmacy, Addis Ababa University, were used. Animals were acclimatized for one week to the experimental environment before the actual experiment. They were housed in polypropylene cages and maintained at 12-hour light-dark cycle. Animals were provided with a commercial food and water *ad libitum*. All procedures and techniques used in this study were in accordance with internationally accepted guideline [21].

### 2.2. Collection of Experimental Plant

The ripened fruits of *S. molle* were collected from Sasiga District, western Ethiopia, where it has an established traditional claim for the treatment of malaria. Identification and authentication of the plant was done by a taxonomist at the National Herbarium, College of Natural and Computational Sciences, Addis Ababa University, where a voucher specimen was coded (GH 01/2017) and deposited for future reference.

### 2.3. Preparation of Crude Extracts

The collected ripened fruits of the plant were thoroughly washed with tap water and cleaned with gauze to remove debris. Then, the fruits were air-dried under shade, and the covering was removed by meshing. The resulting woody seeds were pulverized using mortar and pestle to get a coarse powder (1050 g). Then, the powder was separated into 2 parts: 300 g for aqueous extraction and the other 750 g of the powder for 80% methanol extraction. For both extractions, a mechanical shaker (Bibby Scientific Limited, Stone, Staffordshire, UK) was employed to facilitate the extraction process.

The aqueous extract was prepared as described by Yacob et al. [22]. Pulverized seeds (150 mg) were soaked in 1500 ml of distilled water using an Erlenmeyer flask and the extraction process was facilitated with occasional stirring at 120 rpm for 72 hours. The resulting distillate containing the aqueous crude extract was then separated from the marc with gauze and further filtered by Whatman filter paper number 1 (Whatman®, England) under suction filtration. The same procedure was done for the other 150 mg of pulverized seeds in 1500 ml of distilled water. The extracts from both the procedures were then combined in a round bottom flask and deep-freezed at−27°C and lyophilized (Operon, Korea Vacuum Limited, Korea) for one week to yield a solid residue.

Methanol extraction was done according to Bantie et al. [23] with slight modification. Accordingly, 150 mg of coarse powder was soaked in 1500 ml of 80% methanol in an Erlenmeyer flask. After 72 hours, the resulting distillate containing the 80% methanol crude extract was separated from the marc with gauze and further filtered by Whatman filter paper under suction filtration. The same procedure was done for the left 600 mg of the coarse powder extracting 150 mg at a time. Rotary evaporator (Buchi Rotavapor R-200, Switzerland) and a lyophilizer were used to remove methanol and concentrate the extract, respectively. Finally, a total of 84 g (11.20% yield) and 24 g (8.00% yield) of methanol and aqueous dry extracts were harvested, respectively, and the dried extracts were kept at −20°C until use.

### 2.4. Fractionation of 80% Methanol Crude Extract

The extract with a better antimalarial effect (methanolic extract) was subjected to fractionation using solvents of differing polarity (chloroform, *n*-butanol, and water). Accordingly, 42 grams of the 80% methanol crude extract was suspended in 350 ml of distilled water using a separatory funnel. Then, the suspension was successively shaken with 100 ml of chloroform and *n*-butanol. All the dried fractions were transferred into an amber glass bottle and stored at −20°C, until use. The percentage yield of chloroform, butanol, and aqueous fractions was 45.52%, 35.21%, and 18.27%, respectively.

### 2.5. Acute Toxicity Test

Oral acute toxicity test was performed according to OECD no. 425 guideline [24]. First, 5 overnight fasted female mice, one for each crude extract and fraction, was weighed and dosed with 2000 mg/kg of test substance, given via oral gavage as a single dose. Food was then withheld for further 2 hours. Then, each mouse was observed over a period of 24 hours. Since no death was observed, the same dose was given for four female mice and they were observed for gross behavioral changes such as loss of appetite, hair erection, lacrimation, tremors, convulsions, and mortality for a period of 14 days.

### 2.6. *In Vivo* Antimalarial Screening

#### 2.6.1. Grouping and Dosing of Animals

For screening the *in vivo* antimalarial activity of crude seed extracts and three solvent fractions of *S. molle*, infected male Swiss albino mice were used. Mice were randomly assigned to five groups of 6 mice per group, separately for each crude extract and fraction. The first three groups for each crude extract and fraction received 100 mg/kg (G-I), 200 mg/kg (G-II), and 400 mg/kg (G-III) doses of the treatment, daily for four consecutive days. Dose selection was made based on the result of acute toxicity and preliminary study conducted on the extracts. The remaining two groups for each crude extract and fraction served as negative control (G-IV) and were provided with solvents for reconstitution (Tween 80 2% v/v for the chloroform and butanol fraction and methanol extract or distilled water for the aqueous fraction and extract) (CON), while positive control (G-V) received 10 mg/kg of chloroquine phosphate (CQ).

#### 2.6.2. Four-Day Suppressive Test

Chloroquine-sensitive *P. berghei* ANKA strain was obtained from Ethiopian Public Health Institute (EPHI). The parasites were then maintained by serial passage of blood from infected mice to noninfected ones every week until 30–37% of parasitemia level was attained [25]. Mouse with the aforementioned level of rising parasitemia was used as a donor.

*In vivo* antimalarial activity of the crude extracts and solvent fractions against early chloroquine-sensitive *P. berghei* infection was then carried out according to the 4-day suppressive test described by Peter et al. [26]. First, the parasitemia level of the donor mice was determined from the blood collected by cutting a 0.5 to 1 mm section from tail of the mice with scissors. Donor mice were thus sacrificed and blood collected by cardiac puncture into a falcon tube containing 2% trisodium citrate as anticoagulant [23, 27]. The collected blood samples from all donor mice were pooled together to avoid variability and then diluted in normal saline [23]. The dilution was made based on parasitemia of the donor mice and RBC count of the normal mice in such a way that 1 ml blood contains 5 × 10^7^ infected RBCs [28].

On the first day (D0), each apparently healthy mouse was infected intraperitoneally with 0.2 ml of blood containing about 1 × 10^7^ *P. berghei*-infected RBCs. Then, treatment was started 3 hrs after inoculation and continued daily for three consecutive days (D1–D3). On the 5^th^ day (D4), parameters detailed below were determined and mice were monitored daily for 30 days to determine survival time [26].

#### 2.6.3. Determination of Parasitemia and Survival Time

On D4, blood was collected from the tail of each mouse using clean and nongreasy slides to prepare thin films. After being allowed to air-dry, slides were viewed microscopically using the ×100 objective. The percentage parasitemia (PP) was obtained by counting the number of parasitized RBCs (PRBCs) out of erythrocytes in random fields of the microscope. Two stained slides for each mouse were examined. Three fields with approximately 200–500 cells were counted for each slide and PP for each mouse was determined using the following formula [29–31]:

(1) $$\begin{array}{c} \text{PP}=\frac{\text{PRBC}}{\text{total number of RBCs counted}}\times 100. \end{array}$$

The mean percentage parasitemia suppression (PPS) was calculated using the formula described below [29, 30]:

(2) $$\begin{array}{c} \text{PPS}=\frac{\left(\text{mean PP in negative control}-\text{mean PP in treatment group}\right)}{\text{mean PP in negative control}}\times 100\text{.} \end{array}$$

The mean survival time (MST) for each group was calculated as follows [28]:

(3) $$\begin{array}{c} \text{MST}=\frac{\text{sum of survival time of all mice in a group }\left(\text{days}\right)}{\text{total number of mice in that group}}. \end{array}$$

#### 2.6.4. Determination of Weight, Temperature, and Packed Cell Volume

Weight and rectal temperature of each mouse were recorded just before treatment and after treatment on D4. The mean percentage changes were then calculated and analyzed for each group [26]. In the same way, packed cell volume (PCV) was measured before and after treatment. To determine PCV, blood was collected from the tail of each mouse in heparinized microhematocrit capillary tubes. The capillary tubes were filled to 3/4^th^ of their height with blood and sealed with sealing clay at their dry end. The tubes were then placed on a microhematocrit centrifuge (Centurion Scientific, UK) with the sealed end facing the periphery and centrifuged at 11,000 rpm for 5 minutes [23]. Finally, the tubes were taken out of the centrifuge and PCV was determined using the standard hematocrit reader (Hawksley and Sons, England) according to the formula indicated below [31, 32]:

(4) $$\begin{array}{c} \text{PCV}=\frac{\text{volume of erythrocyte in a given volume of blood}}{\text{total blood volume examined}}\times 100\text{.} \end{array}$$

### 2.7. Phytochemical Screening

Crude seed extracts and solvent fractions of *S. molle* were screened for the presence of major phytochemical constituents. Accordingly, tests for alkaloids, saponins, tannins, flavonoids, phenols, cardiac glycosides, steroids, and terpenoids were performed following standard procedures as described below [33, 34].

### 2.8. Data Analysis

The collected data were organized, entered, and analyzed using SPSS version 22. One-way analysis of variance (ANOVA) followed by Tukey's post-hoc test was used to compare the mean PPS, MST, changes in mean body weight, PCV, and rectal temperature of the *P. berghei*-infected mice between the test groups and the respective control and among different test groups in each test. The analysis at 95% confidence interval and *p* value less than 0.05 was considered to be statistically significant.

## 3. Results

### 3.1. Acute Toxicity Test

Acute toxicity test revealed that no mortality was observed within the first 24 h and the next 14 days of the observation period. The gross behavioral and physical observation of the experimental mice, furthermore, indicated that the plant caused no visible signs of acute toxicity.

### 3.2. 4-Day Suppressive Test

#### 3.2.1. Effect on Parasitemia and Survival Time

*(1) Crude Extract*. The chemosuppressive effect of the plant is summarized in Table 1. All dose levels of each crude seed extract evaluated in the study exhibited a statistically significant (*p* < 0.05) difference in reducing parasite load compared to negative control. The highest percentage of parasitemia inhibition (66.91%) was exhibited by 80% methanol extract at 400 mg/kg/day dose. Nevertheless, the effect produced by the crude extracts was inferior to the standard drug, which cleared the parasite to undetectable level. The 80% methanol crude seed extract of *S. molle* exhibited the longest mean survival time (13.83 days) at the highest dose given.

*(2) Fractions*. The solvent-fraction-treated group revealed a significant (*p* < 0.05) reduction of parasite load compared to negative controls at all doses in a dose-dependent fashion. The highest inhibition (55.60%), however, was exhibited by the chloroform fraction. Chloroform and butanol fractions were able to significantly (*p* < 0.05) prolong the survival time in a dose-dependent manner (Table 1). In all experiments, CQ-treated groups survived throughout the monitoring period (>30 days).

#### 3.2.2. Effect on Body Weight and Rectal Temperature

*(1) Crude Extracts*. The three doses of methanol fraction and highest dose of aqueous fraction (600 mg/kg) significantly (*p* < 0.05) averted body weight loss compared to the negative control. Moreover, all dose levels of the hydromethanolic extract and two dose levels of aqueous extracts (200 mg/kg and 400 mg/kg) were able to significantly prevent body temperature dropping due to parasite infection compared to those in the vehicle-treated groups (Table 2).

*(2) Fractions*. Compared to the negative control, all dose levels of chloroform fractions and butanol fraction as well as highest dose of aqueous fraction exhibited a significant (*p* < 0.05) and dose-dependent protection against body weight loss and rectal temperature dropping. The highest protection in both weight and temperature reduction was exhibited by the chloroform fraction at the highest dose given, 400 mg/kg/day (Table 2).

#### 3.2.3. Effect on Packed Cell Volume

**Figure 1:**
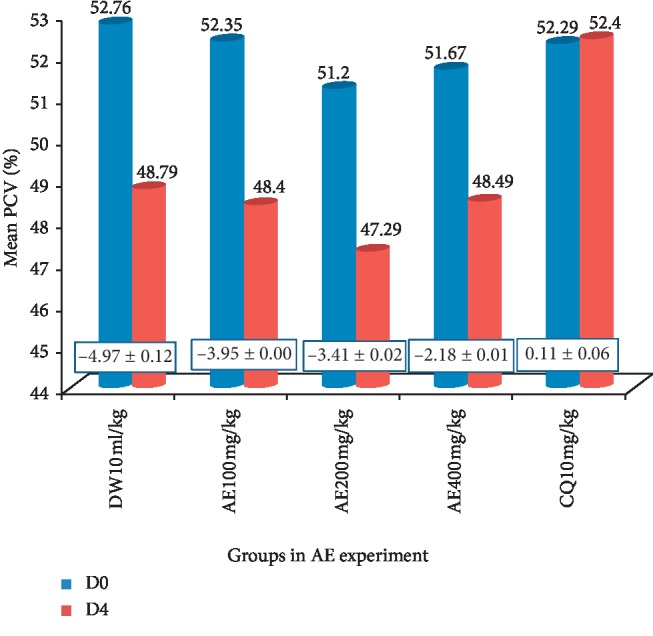
The effect of aqueous crude seed extract of *S. molle* on packed cell volume of *P. berghei*-infected mice.

**Figure 2:**
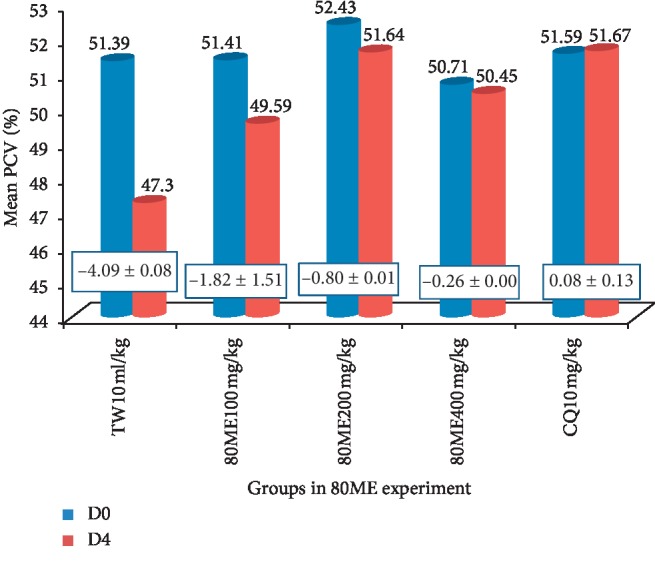
Effect of 80% methanol crude seed extract of *S. molle* on packed cell volume of *P. berghei*-infected mice. Data are expressed as mean ± SEM (*n* = 6); the difference in mean change in PCV was significant at *p* < 0.05; DW: distilled water; TW: 2% Tween 80; CQ: chloroquine base; 80ME: 80% methanol extract; AE: aqueous extract; PCV: packed cell volume; D0: pretreatment value on day 0; and D4: posttreatment value on day four. The numbers in rectangles between the graphs show the change in mean PCV between D0 and D4.

*(1) Crude Extracts*. Significant protection against reduction of PCV (*p* < 0.05) was exhibited in both the aqueous and 80% methanol crude extracts compared to their respective negative controls. The highest protection against reduction of PCV was exhibited by 80% methanol crude seed extract at 400 mg/kg/day (Figure 1), while the lowest protection was exhibited by aqueous crude seed extract at 100 mg/kg/day (Figure 2).

**Figure 3:**
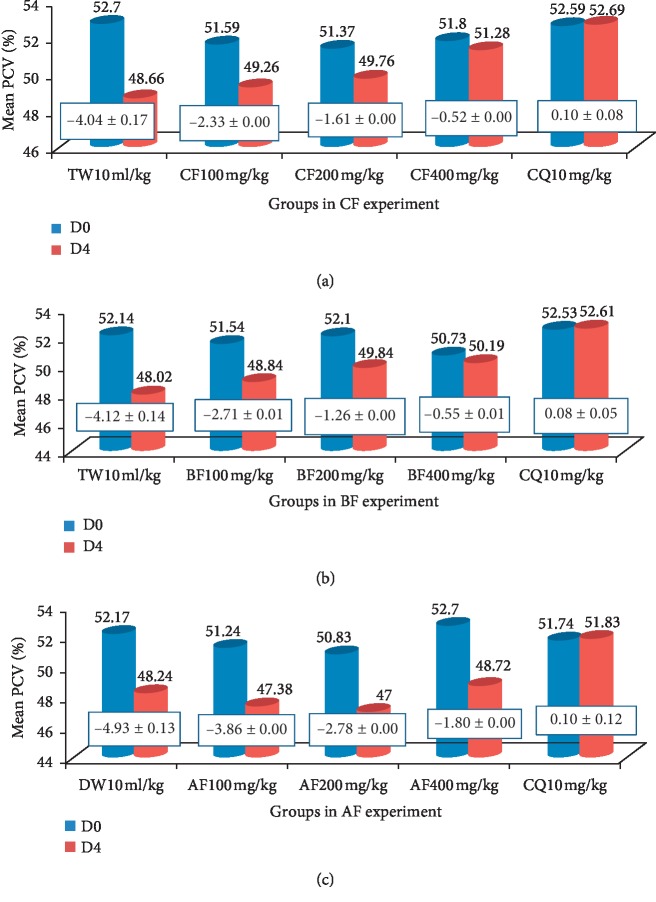
The effect of solvent fraction of *S. molle* (A = CF, B = BF, and C = AF) on packed cell volume of *P. berghei*-infected mice. Data are expressed as mean ± SEM (*n* = 6); TW: negative control, 2% Tween 80; DW: distilled water CQ, positive control, chloroquine base; CF: chloroform fraction; BF: butanol fraction; AF: aqueous fraction; PCV: packed cell volume; D0: pretreatment value on day 0; and D4: posttreatment value on day four. The numbers in rectangles between graphs show the change in mean PCV between D0 and D4.

*(2) Fractions*. Fractions protected infection-induced reduction in PCV. The highest protection was exhibited by the chloroform fraction of the 80% methanol crude seed extract at the highest dose given, 400 mg/kg/day (Figure 3), while the least protection among the three fractions was exhibited by the aqueous fraction at the least dose given, 100 mg/kg/day (Figures 3(a)–3(c)). The rank order of protection from infection-induced reduction in PCV was 80% methanol crude extract > chloroform fraction > butanol fraction > aqueous extract > aqueous fraction.

### 3.3. Phytochemical Screening

Phytochemical screening tests revealed that 80% methanol crude seed extract was positive for alkaloids, saponins, tannins, flavonoids, phenols, cardiac glycosides, steroids, and terpenoids. The least types of secondary metabolites were found in aqueous fraction, which was positive only for alkaloids, tannins, and phenols (Table 3).

## 4. Discussion

Acute toxicity test suggested that the oral medial lethal dose (LD50) of the extracts and fractions could be greater than 2000 mg/kg [24]. This could justify the safe folkloric use of the seed of *S. molle* for the treatment of malaria by the local people in Ethiopia. Previous study [21], furthermore, reported the *in vitro* antimalarial effect of the plant. However, *in vivo* studies take into account prodrug effect and the role of immune system in controlling malaria infection unlike *in vitro* ones [30]. Therefore, to confirm the claimed antimalarial activities of the plant, a 4-day suppressive *in vivo* test was employed in the present study.

The finding of the study evidenced the significant inhibition of parasitemia by both the aqueous and 80% methanol crude seed extracts and all the three fractions of the seed of *S. molle*. The 80% methanol crude extract and aqueous fraction exhibited the maximum and minimum chemosuppression effect, respectively. This is in agreement with a similar study done on *Croton macrostachyus* by Bantie et al. [23]. On the contrary, a study conducted by Fantahun et al. [35] on *Strychnos mitis* reported the highest antimalarial effect of its aqueous crude extract.

Among the solvent fractions evaluated, the highest parasite inhibition was exhibited by the chloroform fraction. This finding is consistent with other studies [28, 36], in which the chloroform fractions showed higher chemosuppressive activity than the aqueous fraction. This suggests that the active bioactive agents are concentrated in nonpolar fraction.

The bioactive secondary metabolites revealed in phytochemical analysis could be responsible for the antimalarial activity of the plant [23]. The terpenoids, phenolic compounds, and flavonoids observed in this plant have been proved to possess potential immunomodulatory, anti-inflammatory, and antioxidant activity [37, 38]. Therefore, the chemosuppressive effect could be via indirect boosting of the immune system or inhibition of other target pathways which are not fully realized [32].

An ideal antimalarial agents derived from plants are expected to prevent reduction in PCV, body weight loss, and reduction in body temperature due to the development of parasitemia [29]. Even though a significant prevention of body weight loss was exhibited by crude extracts and the solvent fractions compared to negative control, the mean value of the body weight showed reduction in treatment groups. Similarly, PCV was measured to evaluate the effectiveness of the plant in ablating malaria-induced hemolysis. Chloroform fraction exhibited the maximal protection of hemolysis compared to the two other fractions. The mean value of the PCV and body weight, however, showed reduction in mice treated with the crude extracts and fractions on day 4 as compared to day 0. These could be attributed to the inability of the extracts and fractions to completely clear the parasites from the bodies of the mice other than reduction to different levels [28, 32].

The three doses of methanolic extract and the middle and higher doses of aqueous extract of tested plant and all doses of the chloroform and butanol fractions significantly prevented rectal temperature dropping due to parasitemia. These activities probably indicate that the extracts ameliorate some pathological processes that cause reduction in internal body temperature and metabolic rates [23, 31].

If antimalarial activity of a compound displayed a percent growth inhibition of >50% at a dose of 500–250, 250–100, and <100 mg/kg/day, the literature grades it as moderate, good, and very good, respectively [27, 39]. Therefore, the seed of *S. molle* has a good antimalarial activity. However, the aqueous fraction is found to be inactive as its parasite suppression was less than 30% even at the highest dose administered.

## 5. Conclusion

Acute toxicity test conducted on *S. molle* confirmed the safety of the plant up to a dose of 2000 mg/kg. Furthermore, the findings of the present study indicate that the seed of *Schinus molle* has significant *in vivo* antimalarial activity. The highest antimalarial effect was exhibited by the 80% methanol crude extract at the highest dose tested. Among the fractions tested, the chloroform fraction was found to be the most active in suppressing the parasite indicating the possible localization of the active compounds in this fraction. Moreover, the data would provide evidence to uphold the earlier *in vitro* antimalarial investigation on the plant as well as the traditional use of the plant by the local communities for the treatment of malaria in Ethiopia. Consequently, the seed of *Schinus molle* could be used as a potential source to develop more effective and safer antimalarial drugs.

## Figures and Tables

**Table 1:** The effect of crude seed extracts and solvent fractions of *S. molle* on parasitemia and survival time of *P. berghei*-infected mice.

Group	% parasitemia	% suppression	Survival time (day)
80ME100	33.01 ± 0.92	35.72^a^*∗*^,c^*∗*^,d^*∗*^,e^*∗*^^	9.50 ± 0.43^a^*∗*^,d^*∗*^,e^*∗*^^
80ME200	22.75 ± 0.83	55.70^a^*∗*^,b^*∗*^,d^*∗*^,e^*∗*^^	10.17 ± 0.48^a^*∗*^,d^*∗*^,e^*∗*^^
80ME400	16.99 ± 0.73	66.91^a^*∗*^,b^*∗*^,c^*∗*^,e^*∗*^^	13.83 ± 0.87^a^*∗*^,b^*∗*^,c^*∗*^,e^*∗*^^
CON	51.35 ± 1.66	0.00	6.33 ± 0.49
CQ10	0.00 ± 0.00	100.00^a^*∗*^^	>30.00 ± 0.00^a^*∗*^^

AE100	38.00 ± 1.32	27.18^a^*∗*^,c^*∗*^,d^*∗*^,e^*∗*^^	6.50 ± 0.34^d^*∗*^,e^*∗*^^
AE200	35.42 ± 1.20	32.15^a^*∗*^,b^*∗*^,d^*∗*^,e^*∗*^^	6.67 ± 0.33^d^*∗*^,e^*∗*^^
AE400	33.89 ± 1.32	35.08^a^*∗*^,b^*∗*^,c^*∗*^,e^*∗*^^	8.00 ± 0.37^a^*∗*^,b^*∗*^,c^*∗*^,e^*∗*^^
CON	52.20 ± 2.20	0.00	6.33 ± 0.21
CQ10	0.00 ± 0.00	100.00^a^*∗*^^	>30.00 ± 0.00^a^*∗*^^

CF100	37.52 ± 1.10	32.69^a^*∗*^,c^*∗*^,d^*∗*^,e^*∗*^^	8.33 ± 0.33^a^*∗*^,d^*∗*^,e^*∗*^^
CF200	34.02 ± 0.93	38.97^a^*∗*^,b^*∗*^,d^*∗*^,e^*∗*^^	9.17 ± 0.31^a^*∗*^,d^*∗*^,e^*∗*^^
CF400	24.75 ± 1.37	55.60^a^*∗*^,b^*∗*^,c^*∗*^,e^*∗*^^	12.17 ± 0.48^a^*∗*^,b^*∗*^,c^*∗*^,e^*∗*^^
CON	55.74 ± 1.06	0.00	6.33 ± 0.42
CQ10	0.00 ± 0.00	100.00^a^*∗*^^	>30.00 ± 0.00^a^*∗*^^

BF100	36.23 ± 1.32	31.49^a^*∗*^,c^*∗*^,d^*∗*^,e^*∗*^^	8.17 ± 0.40^a^*∗*^,d^*∗*^,e^*∗*^^
BF200	33.00 ± 1.20	37.59^a^*∗*^,b^*∗*^,d^*∗*^,e^*∗*^^	8.67 ± 0.49^a^*∗*^,d^*∗*^,e^*∗*^^
BF400	29.28 ± 1.67	44.63^a^*∗*^,b^*∗*^,c^*∗*^,e^*∗*^^	10.67 ± 0.67^a^*∗*^,b^*∗*^,c^*∗*^,e^*∗*^^
CON	52.88 ± 2.20	0.00	6.17 ± 0.31
CQ10	0.00 ± 0.00	100.00^a^*∗*^^	>30.00 ± 0.00^a^*∗*^^

AF100	43.52 ± 0.92	15.64^a^*∗*^,c^*∗*^,d^*∗*^,e^*∗*^^	6.50 ± 0.43^e^*∗*^^
AF200	41.51 ± 1.33	19.54^a^*∗*^,b^*∗*^,d^*∗*^,e^*∗*^^	6.83 ± 0.48^e^*∗*^^
AF400	38.05 ± 1.59	26.25^a^*∗*^,b^*∗*^,c^*∗*^,e^*∗*^^	7.33 ± 0.49^e^*∗*^^
CON	51.59 ± 1.64	0.00	6.33 ± 0.33
CQ10	0.00 ± 0.00	100.00^a^*∗*^^	>30.00 ± 0.00^a^*∗*^^

Data are expressed as mean ± SEM (*n* = 6); ^a^compared to either CON; ^b^compared to 100 mg/kg; ^c^compared to 200 mg/kg; ^d^compared to 400 mg/kg; ^e^compared to CQ10; and ^*∗*^*p* < 0.05. AE = aqueous crude extract, 80ME = 80% methanol crude extract, CF = chloroform fraction, BF = butanol fraction, AF = aqueous fraction, CON = control, and CQ = chloroquine base. Numbers after letters in the first column refer to dose in mg/kg.

**Table 2:** Body weight and rectal temperature changes of *P. berghei*-infected mice treated with the crude seed extracts and solvent fractions of *S. molle*.

Group	Weight (g)			Temperature (°C)		
	D0	D4	Change	D0	D4	Change
80ME100	28.50 ± 0.63	26.47 ± 0.68	−2.03 ± 0.17^a^*∗*^c^*∗*^d^*∗*^e^*∗*^^	36.70 ± 0.35	35.50 ± 0.36	−1.20 ± 0.02^a^*∗*^c^*∗*^d^*∗*^e^*∗*^^
80ME200	28.66 ± 0.66	27.53 ± 0.69	−1.13 ± 0.13^a^*∗*^b^*∗*^d^*∗*^e^*∗*^^	37.05 ± 0.32	36.52 ± 0.32	−0.53 ± 0.01^a^*∗*^b^*∗*^d^*∗*^e^*∗*^^
80ME400	28.63 ± 0.66	28.42 ± 0.66	−0.21 ± 0.017^a^*∗*^b^*∗*^c^*∗*^e^*∗*^^	36.94 ± 0.27	36.70 ± 0.27	−0.24 ± 0.01^a^*∗*^b^*∗*^c^*∗*^e^*∗*^^
CON	28.17 ± 0.82	24.54 ± 0.50	−3.63 ± 1.46	36.85 ± 0.22	34.47 ± 0.27	−2.38 ± 0.06
CQ10	28.52 ± 0.79	28.80 ± 0.83	0.28 ± ^a^*∗*^^	37.20 ± 0.22	38.00 ± 0.21	0.80 ± 0.03^a^*∗*^^

AE100	28.14 ± 0.77	24.68 ± 0.77	−3.46 ± 0.64^d^*∗*^e^*∗*^^	37.15 ± 0.44	34.78 ± 0.43	−2.37 ± 0.01^c∗d∗e∗^
AE200	28.35 ± 0.55	24.89 ± 0.55	−3.45 ± 0.01^d^*∗*^e^*∗*^^	36.88 ± 0.22	34.54 ± 0.24	−2.34 ± 0.00^a∗b∗d∗e∗^
AE400	28.54 ± 0.86	25.38 ± 0.84	−3.16 ± 0.04^a^*∗*^b^*∗*^c^*∗*^e^*∗*^^	36.97 ± 0.30	35.12 ± 0.29	−1.85 ± 0.01^a^*∗*^b^*∗*^c^*∗*^e^*∗*^^
CON	28.22 ± 0.85	24.74 ± 0.88	−3.48 ± 0.10	37.05 ± 0.42	34.69 ± 0.41	−2.36 ± 0.04
CQ10	28.23 ± 0.50	28.45 ± 0.46	0.22 ± 0.057^a^*∗*^^	36.99 ± 0.28	37.67 ± 0.27	0.68 ± 0.04^a^*∗*^^

CF100	28.44 ± 0.80	26.15 ± 0.79	−2.29 ± 0.01^a^*∗*^c^*∗*^d^*∗*^e^*∗*^^	36.95 ± 0.16	35.29 ± 0.15	−1.66 ± 0.01^a^*∗*^c^*∗*^d^*∗*^e^*∗*^^
CF200	28.05 ± 0.68	26.63 ± 0.69	−1.42 ± 0.02^a^*∗*^b^*∗*^d^*∗*^e^*∗*^^	36.99 ± 0.29	36.15 ± 0.30	−0.84 ± 0.00^a^*∗*^b^*∗*^d^*∗*^e^*∗*^^
CF400	28.18 ± 0.64	27.64 ± 0.63	−0.54 ± 0.01^a^*∗*^b^*∗*^c^*∗*^e^*∗*^^	36.89 ± 0.29	36.39 ± 0.27	−0.50 ± 0.01^a^*∗*^b^*∗*^c^*∗*^e^*∗*^^
CON	28.41 ± 0.86	24.82 ± 0.57	−3.59 ± 0.85	36.81 ± 0.35	34.48 ± 0.32	−2.33 ± 0.06
CQ10	28.59 ± 0.88	28.83 ± 0.86	0.24 ± 0.04^a^*∗*^^	37.02 ± 0.32	37.71 ± 0.29	0.70 ± 0.05^a^*∗*^^

BF100	28.28 ± 0.39	24.90 ± 0.66	−3.39 ± 0.08^a^*∗*^c^*∗*^d^*∗*^e^*∗*^^	37.02 ± 0.67	35.04 ± 0.66	−1.98 ± 0.09^a^*∗*^c^*∗*^d^*∗*^e^*∗*^^
BF200	29.12 ± 0.66	26.47 ± 0.67	−2.65 ± 0.02^a^*∗*^b^*∗*^d^*∗*^e^*∗*^^	36.87 ± 0.66	35.74 ± 0.67	−1.14 ± 0.01^a^*∗*^b^*∗*^d^*∗*^e^*∗*^^
BF400	28.08 ± 0.69	26.65 ± 0.70	−1.43 ± 0.01^a^*∗*^b^*∗*^c^*∗*^e^*∗*^^	37.01 ± 0.69	36.26 ± 0.70	−0.75 ± 0.01^a^*∗*^b^*∗*^c^*∗*^e^*∗*^^
CON	27.93 ± 0.81	24.28 ± 0.79	−3.65 ± 0.09	36.97 ± 0.81	34.66 ± 0.80	−2.31 ± 0.08
CQ10	28.24 ± 0.61	28.43 ± 0.60	0.19 ± .03^a^*∗*^^	36.92 ± 0.62	37.54 ± 0.60	0.62 ± 0.05^a^*∗*^^

AF100	28.33 ± 0.68	24.72 ± 0.67	−3.62^d^*∗*^e^*∗*^^	36.98 ± 0.26	34.66 ± 0.26	−2.32 ± 0.01^d^*∗*^e^*∗*^^
AF200	28.34 ± 0.57	24.75 ± 0.56	−3.59^d^*∗*^e^*∗*^^	36.96 ± 0.26	34.61 ± 0.27	−2.35 ± 0.02^d^*∗*^e^*∗*^^
AF400	28.05 ± 0.79	24.82 ± 0.77	−3.2301^a^*∗*^b^*∗*^c^*∗*^e^*∗*^^	37.04 ± 0.25	34.79 ± 0.24	−2.25 ± 0.01^a^*∗*^b^*∗*^c^*∗*^e^*∗*^^
CON	28.51 ± 0.75	24.86 ± 0.81	−3.65	37.05 ± 0.27	34.72 ± 0.34	−2.33 ± 0.10
CQ10	28.45 ± 0.67	28.68 ± 0.68	0.22^a^*∗*^^	36.89 ± 0.22	37.56 ± 0.21	0.67 ± 0.06^a^*∗*^^

Data are expressed as mean ± SEM (*n* = 6); ^a^compared to CON; ^b^compared to 100 mg/kg; ^c^compared to 200 mg/kg; ^d^compared to 400 mg/kg; ^e^compared to CQ10; and^*∗*^*p* < 0.05. D0 = pretreatment value on day 0, D4 = posttreatment value on day four. AE = aqueous crude extract, 80ME = 80% methanol crude extract, CF = chloroform fraction, BF = butanol fraction, AF = aqueous fraction, CON = control, and CQ = chloroquine base. Numbers after letters in the first column refer to dose in mg/kg.

**Table 3:** Phytoconstituents of crude extracts and solvent fractions of the seed of *S. molle*.

Phytochemicals	80% methanol crude extract	Aqueous crude extract	Chloroform fraction	Butanol fraction	Aqueous fraction
Alkaloids	+	+	+	+	+
Tannins	+	+	+	+	+
Saponins	+	+	+	+	−
Flavonoids	+	+	+	+	−
Terpenoids	+	−	+	−	−
Steroids	+	−	+	−	−
Phenols	+	+	−	+	+
Glycosides	+	−	−	+	−

− indicates absence; + indicates presence of corresponding phytochemical constituent.

## Data Availability

Vouchers and specimens of the experimental plant used for this study are deposited at the National Herbarium of Addis Ababa University, College of Natural Sciences. The datasets supporting the conclusion of this study are available from the corresponding author upon reasonable request.
